# Dehydrocorydaline Accelerates Palatal Wound Healing in Mice Through Suppression of the p38 MAPK/CCL2 Axis and Macrophage Chemotaxis: A Preliminary Study

**DOI:** 10.3390/biomedicines14091918

**Published:** 2026-08-27

**Authors:** Yingyi Chen, Zhaona Liu, Yijia Wang, Guiyang Xia, Yitong Liu, Huan Xia, Minfeng Wang, Sheng Lin, Yi Liu

**Affiliations:** 1 Beijing Key Laboratory of Tooth Regeneration and Function Reconstruction, Laboratory of Tissue Regeneration and Immunology, Department of Periodontics, School of Stomatology, Capital Medical University, No. 9 Fanjiacun Road, Beijing 100070, China; chenyingyi15@163.com (Y.C.); liuzhaona1921@163.com (Z.L.); yijiawang1994@163.com (Y.W.); yitongliu@mail.ccmu.edu.cn (Y.L.); kitty7596@sina.com (M.W.); 2 Department of Stomatology, Longfu Hospital, Beijing 100010, China; 3 Key Laboratory of Chinese Internal Medicine, Ministry of Education and Beijing Municipality, Dongzhimen Hospital, Beijing University of Chinese Medicine, Beijing 100700, China; nyxiaguiyang@163.com (G.X.); 15801477748@163.com (H.X.)

**Keywords:** dehydrocorydaline, macrophage, chemotaxis, CCL2

## Abstract

**Background/Objectives:** Excessive inflammation is a critical contributor to impaired oral mucosal wound healing, yet effective therapeutic strategies are still lacking. Although dehydrocorydaline (DHC) has been reported to exhibit anti-inflammatory and analgesic properties, its role in wound healing and the underlying mechanisms have not been fully elucidated. This study aimed to investigate whether DHC accelerates palatal wound healing and to elucidate the role of the p38 mitogen-activated protein kinase (MAPK)/CCL2 signaling axis in DHC-mediated regulation of macrophage chemotaxis. **Methods:** *In vitro*, macrophages were stimulated with 1 μg/mL lipopolysaccharide (LPS) and treated with DHC at 0.1, 1, and 10 μM. The chemotactic response and inflammatory function of macrophages were assessed using real-time PCR, ELISA, Western blotting, and Transwell assays. Molecular docking simulations and Western blotting analyses were performed to examine the regulatory effect of DHC on MAPK signaling pathway. *In vivo*, a full-thickness palatal mucoperiosteal wound extending from the left maxillary first to third molars was established in mice by scalpel scraping. The effects of topical 10 μM DHC gel on wound healing were evaluated using stereomicroscopy, histological staining, and real-time PCR at 0, 3, and 5 days post-modeling. **Results:** *In vitro*, DHC effectively downregulated the expression of chemokines, including C-C motif chemokine ligand 2 (*Ccl2*), *Ccl5*, *Ccl22*, C-X-C motif chemokine ligand 10 (*Cxcl10*), and *Ccl24*, with the most significant inhibitory effect on *Ccl2* (70.8% inhibition). In Transwell assays, DHC reduced macrophage migration by 68.2%. Mechanistically, DHC prominently inhibited the activation of the MAPK signaling pathway. *In vivo*, DHC treatment accelerated wound healing and markedly reduced macrophage infiltration in mouse palatal wound tissues. **Conclusions:** These findings demonstrated that DHC accelerated palatal wound healing 1.6-fold in mice. DHC suppressed macrophage chemotaxis by 68.2% through modulation of the MAPK signaling pathway.

## 1. Introduction

The oral mucosa functions as a critical barrier against physical and chemical irritants and pathogenic microbial invasion [1]. Oral mucosal damage can arise from diverse etiologies, including trauma, invasive dental procedures, mucosal diseases, and head/neck radiotherapy [1]. Wound healing is a tightly regulated biological process encompassing four sequential stages: hemostasis, inflammation, proliferation, and remodeling [2]. Dysregulation of this process causes not only chronic nonhealing wounds and excessive scarring but also severe pain that impairs nutritional intake and oral function [3]. Additionally, the oral complex microbial ecosystem increases susceptibility to infection following mucosal injury [4].

Wound healing involves multiple stages coordinated by precise cellular and molecular interactions [5]. In response to chemokine signals, inflammatory cells infiltrate the wound site to eliminate debris and pathogens [6]. Macrophages play a central role in orchestrating the inflammatory phase through activation and polarization upon recruitment to the wound [7]. These activated states are broadly categorized as M1 and M2 phenotypes [8]. Studies indicate that prolonged inflammation is associated with impaired wound healing or excessive fibrosis [9]. During the initial inflammatory phase, M1 macrophages predominate, producing pro-inflammatory factors to clear pathogens and debris [10]. Pro-inflammatory M1 macrophages secrete cytokines (e.g., IL-1β, IL-6, TNF-α, iNOS) and chemokines (e.g., CCL2, CCL5, CXCL2, CXCL12) [11], which further recruit macrophages and initiate inflammatory cascades [12]. Excessive inflammation can hinder wound healing and enhance scar formation [5]. Current clinical management of oral mucosal wounds remains largely limited to symptomatic treatments such as pain control, infection prevention, and general supportive care. Effective therapeutic strategies that target excessive macrophage-mediated inflammation are still lacking. Given that chemokine environment imbalance may be a key driver of chronic wound progression, targeting chemokines represents a potential therapeutic strategy for wound management [5].

Dehydrocorydaline (DHC) is an isoquinoline alkaloid derived from the tubers and aerial parts of *Corydalis corydalina* (Papaveraceae), with a molecular formula of C_22_H_23_NO_4_^+^ and molecular weight of 365.41 Da [13]. DHC exhibits significant anti-inflammatory properties, with reported efficacy in treating inflammatory pain (e.g., spastic pain, abdominal pain, wound pain) and widespread clinical application [14,15]. It also demonstrates favorable biological safety profiles [14,15]. Ishiguro et al. showed that DHC effectively suppresses inflammatory factor expression in macrophages [16], while Bin et al. confirmed via RNA-seq that DHC significantly regulates the expression of chemotaxis-related and inflammatory factors in macrophages, including *Il1b*, *Ccl5*, *Cxcl10*, *Ccr2*, *Ccr3*, *Ccl22*, *Ccr1*, and *Ccl24* [17]. However, whether DHC can promote palatal wound healing by inhibiting macrophage chemotaxis and inflammatory function remains unclear.

The aim of this study is to investigate the effects of DHC on palatal wound healing and to elucidate the underlying molecular mechanisms. Investigating these aspects could provide evidence for DHC’s potential as a natural small-molecule therapeutic for the treatment of oral mucosal wounds.

## 2. Materials and Methods

### 2.1. Antibodies and Reagents

Primary antibodies used in this study included anti-ERK1/2, anti-phospho-ERK1/2, anti-β-actin, anti-p38, anti-phospho-p38, and anti-iNOS (all from Cell Signaling Technology, Danvers, MA, USA), anti-HSP90 (Santa Cruz Biotechnology, Dallas, TX, USA), anti-F4/80 (Invitrogen, Carlsbad, CA, USA), anti-CCL2, and anti-IL-1β (Abcam, Cambridge, UK). Alexa Fluor 555-conjugated anti-rabbit IgG (Cell Signaling Technology) and Alexa Fluor 488-conjugated goat anti-rat IgG (Abcam) were used as secondary antibodies for immunofluorescence staining. Lipopolysaccharide (LPS) from *Escherichia coli* was purchased from InvivoGen (San Diego, CA, USA), and SB203580 was purchased from MedChemExpress (Monmouth Junction, NJ, USA). ELISA kits for CCL2, TNF-α, and IL-6 were purchased from BioLegend (San Diego, CA, USA). Detailed information on antibodies and reagents, including clone numbers, catalog numbers, and host species, is provided in Table S1.

### 2.2. Extraction and Isolation of DHC

As described in the previous study [18], the methanol extract of *C. yanhusuo* dried tuber was concentrated and dried, separated using chromatography, and eluted through Sephadex LH-20 CC (5 × 120 cm), petroleum ether (PE) -CH_2_C_l2_-MeOH (5:5:1, *v*:*v*:*v*). The active small-molecule compound DHC was separated with a Sephadex LH-20 column, and the MeOH eluent was eluted again and purified using HPLC separation.

### 2.3. Animals

Male C57BL/6J mice were purchased from the Institute of Animal Science at Vital River (Beijing, China) and housed under standardized specific pathogen-free conditions with free access to food and water. All animal experiments followed an approved animal research protocol (KQYY-202206-007). For the in vitro peritoneal macrophage isolation, male C57BL/6J mice aged 8–10 weeks were used. For the in vivo palatal wound healing experiments, male C57BL/6J mice aged 8 weeks were used to ensure age matching across experimental groups. For the palatal wound healing experiments, a total of 24 mice were used (*n* = 4 per group at 0, 3, and 5 days post-treatment). For the macrophage depletion experiments, an additional 24 mice were used (*n* = 4 per group at 0, 3, and 5 days post-treatment). An additional independent palatal wound healing model was established for PCR analysis of *Ccl2* and inflammatory cytokines in wound tissues (day 3, *n* = 3; day 5, *n* = 6).

### 2.4. Isolation of Mouse Peritoneal Macrophages

For peritoneal macrophage isolation, male C57BL/6J mice aged 8–10 weeks were used. Three days after intraperitoneal injection of thioglycolate (Sigma-Aldrich, St. Louis, MO, USA), peritoneal exudate cells were collected by peritoneal lavage. The lavage fluid was centrifuged, cell precipitates were collected, resuspended, and counted, slabs were plated at 2 × 10^6^ cells per well, and cultures were grown for 6–18 h. Peritoneal macrophages were cultured in RPMI medium (Invitrogen, Carlsbad, CA, USA) supplemented with 10% heat-inactivated fetal bovine serum (FBS; Equitech-Bio, Kerrville, TX, USA), 100 U/mL penicillin and 100 μg/mL streptomycin (Invitrogen), as well as 2 mM L-glutamine (Invitrogen). The cells would be stimulated within a week after collection.

### 2.5. Culture of RAW264.7

The RAW264.7 cells were acquired from the China Infrastructure of Cell Line Resources (Beijing, China) and cultured using Dulbecco’s modified Eagle’s medium (DMEM; Invitrogen) supplemented with 10% FBS, 100 U/mL penicillin, 100 μg/mL streptomycin (Invitrogen), and 2 mM L-glutamine (Invitrogen). The cells were maintained at a temperature of 37 °C with a CO_2_ concentration of 5% in a humidified incubator. The culture media were refreshed every two to three days, and cell passages were conducted when the confluency reached approximately 80%.

### 2.6. Treatments

Mouse peritoneal macrophages and RAW264.7 cells were seeded in six-well plates at a density of 2 × 10^6^ cells/well and cultured in an incubator with 5% CO_2_ at 37 °C. All cells were treated for 24 hours with different concentrations of DHC (0.1 μM, 1 μM, and 10  μM) and 1 μg/mL LPS. The control groups were treated with PBS buffer at the same intervention time point.

### 2.7. Cell Viability Assay

Cell viability was assessed using a Cell Counting Kit-8 (CCK-8; Dojindo, Kumamoto, Japan). Briefly, RAW264.7 cells were seeded into 96-well plates at a density of 5 × 10^3^ cells/well and cultured overnight to allow for attachment. Subsequently, the cells were treated with DHC at various concentrations (10 nM, 0.1 μM, 1 μM, and 10 μM) for 24 h. Following the treatment, 10 μL of CCK-8 reagent was added to each well, and the plates were incubated at 37 °C for an additional 2 h in the dark. The optical density (OD) was measured at a wavelength of 450 nm using a microplate reader (Bio-Rad, Hercules, CA, USA). Cell viability (%) was calculated using the following formula: [(OD_experimental_ − OD_blank_)/(OD_control_ − OD_blank_)] × 100%. All assays were performed in quadruplicate.

### 2.8. Quantitative Real-Time PCR Analysis

The Trizol reagent (Invitrogen) was utilized for the extraction of total RNA. cDNA was synthesized from 2 μg of total RNA according to the manufacturer’s instructions. Real-time PCR reactions were conducted using Power SYBR^®^ Green PCR Master Mix (Life Technologies, Warrington, UK) as per the provided guidelines. The primer set employed encompassed *Ccl2*, *Ccl5*, *Ccl22*, *Cxcl10*, *Ccl24*, *Ccr1*, *Ccr2*, *Ccr3*, *Ccr5, Cxcr4, Nos2*, *Tnfa*, *Il1b*, *Il6, Gapdh*, and *Actb*. The above primer sequence information is detailed in Table S2.

### 2.9. Transwell Migration Assay

The influence of DHC on LPS-induced chemotaxis responses was assessed using macrophages. Peritoneal macrophages from mice were isolated using the method described earlier and then cultured overnight in RPMI 1640 medium supplemented with 10% FBS and 1% P/S. Subsequently, a total of 1 × 10^6^ cells were transferred into 100 µL of RPMI medium containing 2% FBS and 1% penicillin/streptomycin, which was loaded into the upper chamber of a Transwell insert (8 µm pore size, Corning, Kaiserslautern, Germany). The filters were placed in the wells (the lower chamber) of various experimental groups. According to the experimental design, the cells were treated with LPS alone, or in combination with DHC (10 µM), CCL2 neutralizing antibody (CCL2 nAb), or the inhibitor SB203580 for 24 h. The experimental group was treated with LPS and DHC at a concentration of 10 µM for 24 h. After incubation at 37 °C with 5% CO_2_ for 24 h, the cells were treated with 95% ethanol for fixation and subjected to staining using a solution containing 0.19% crystal violet. Five fields were randomly collected under a 40× microscope, and cells across the membrane were counted using the ImageJ (version 1.53t) software system.

### 2.10. Enzyme-Linked Immunosorbent Assay (ELISA)

The expression levels of CCL2, IL-6, and TNF-α were measured using uncoated ELISA kits. Absorbance was measured at 450 nm and 570 nm with a Microplate Reader (Bio-Rad). Subsequently, the value at 570 nm was subtracted from the value at 450 nm.

### 2.11. Molecular Docking Simulation

The crystal structure of p38 was retrieved from the RCSB Protein Data Bank. Using Discovery Studio 2019 software, the structure was prepared by removing ligands and water molecules, adding hydrogen atoms, calculating charges, and optimizing amino acids. The structural files of small-molecule compounds were obtained from the PubChem database (National Institutes of Health, USA). Both protein and compound files were converted to PDBQT format. Molecular docking was performed using Discovery Studio 2019, and the results were visualized for analysis.

### 2.12. Western Blot Analysis

Cells were lysed in radioimmunoprecipitation assay buffer (Beyotime, Shanghai, China) and centrifuged to collect supernatants. Total proteins obtained were separated by 7.5% sodium dodecyl sulfate–polyacrylamide gel electrophoresis (Applygen, Beijing, China) and transferred onto Immobilon^TM^-P membranes (Millipore, Billerica, MA, USA). The membranes were blocked with Tris-buffered saline with Tween 20 solution containing 5% skim milk (Sigma-Aldrich) for 1 h at room temperature, then incubated overnight at 4 °C with primary antibodies. After washing, membranes were incubated with horseradish peroxidase-conjugated secondary antibodies (1:2000; Pierce, Malibu, CA, USA) for 1 h at room temperature. Protein bands were visualized using an enhanced chemiluminescence kit (Thermo Fisher Scientific, Waltham, MA, USA) according to the manufacturer’s instructions.

### 2.13. Palatal Mucosal Wound Model in Mice

For the palatal wound healing model, male C57BL/6J mice aged 8 weeks were used. Prior to surgery, each mouse was anesthetized via intraperitoneal injection of a ketamine/xylazine cocktail (80 mg/kg ketamine hydrochloride + 10 mg/kg xylazine; Sigma-Aldrich). The palatal region was disinfected with 75% ethanol. A full-thickness mucosal wound was established on the left side of the palate using a sterile scalpel blade, and the mucoperiosteum was scraped. The wound boundaries are defined as follows: mesial edge of the left maxillary first molar to the mesial edge of the left maxillary third molar. Mice with wounds were randomly divided into two groups: PBS control group and DHC treatment group. To prepare the treatments, 2% methylcellulose gels were formulated by dissolving methylcellulose powder (Sigma-Aldrich) in either PBS (control gel) or a PBS solution containing DHC to achieve a final concentration of 10 μM (treatment gel). Mice in the experimental group received daily topical application of 10 μM DHC gel at the wound site after surgery, while those in the control group were administered an equal volume of PBS gel at the same site. At 0, 3, and 5 days post-treatment, mice were euthanized to evaluate wound healing at different stages (*n* = 4 per group at each time point). The wound healing area was measured using a stereomicroscope (Olympus SZX16, Tokyo, Japan) and ImageJ (version 1.53t) software (*n* = 4 per group).

### 2.14. Histological Staining

Maxillary palates were isolated and fixed in 4% paraformaldehyde for 24 h, followed by decalcification in 14% EDTA buffer. After dehydration, samples were embedded in paraffin. Deparaffinized sections were subjected to hematoxylin and eosin (H&E) staining (*n* = 4 per group at each time point). For assessment of wound collagen deposition, Masson’s trichrome staining was performed using a kit (Solarbio, Beijing, China) according to the manufacturer’s instructions (day 3: *n* = 4 per group; day 5: *n* = 3 per group). Wound width and collagen deposition area were measured with ImageJ (version 1.53t) software.

### 2.15. Immunofluorescence Staining

To evaluate local macrophage infiltration and polarization during the early stage of wound healing, palatal wound tissues from the mice sacrificed on the third day post-operation were selected for immunofluorescence staining (*n* = 3 per group). Tissue sections were baked and dewaxed before being placed in a sodium citrate buffer (0.01 mol/L, pH 6.0, powder) for antigen extraction. All sections were incubated at 37 °C and treated with 10% serum for an additional hour to minimize nonspecific staining. Following this, the sections were subjected to an overnight incubation at 4 °C with primary antibodies F4/80 (1:1000) and iNOS (1:500). Subsequently, a two-hour incubation was carried out using a mixture of Alexa Fluor 488-conjugated goat anti-rat IgG (1:500, Abcam) and Alexa Fluor 555-conjugated anti-rabbit IgG (1:500, Cell Signaling Technology) secondary antibodies. Finally, the sections were counterstained with DAPI and evaluated using a fluorescence microscope (Olympus BX61) to determine F4/80^+^ and iNOS^+^ areas in palatal wound tissue; analysis was performed using ImageJ (version 1.53t) software.

### 2.16. Zebrafish Mechanical Injury Model

Transgenic zebrafish (3 days post-fertilization, 3 dpf) with green fluorescent macrophages were placed in 6-well plates (*n* = 30). Different concentrations of DHC solution (6.25 μM~100 μM) were administered, with a normal control group set. After 5 h of incubation at 28 °C, the maximum tolerated concentration (MTC) of DHC in normal zebrafish was determined to establish a safe dosage range and rule out drug-induced toxicity. Based on this initial screening, the MTC was determined to be 50 μM. Subsequently, to rigorously evaluate the dose-dependent efficacy of DHC within this non-toxic range, 3 dpf transgenic zebrafish were treated with DHC at 12.5 μM (1/4 MTC), 25 μM (1/2 MTC), and 50 μM (1 MTC) (*n* = 9 per group), along with normal control and model control groups. Except for the normal control group, all other experimental groups were subjected to caudal fin amputation via surgery to establish the zebrafish caudal fin injury model. After 5 h of treatment at 28 °C, images of the tail injury sites were captured under a fluorescence microscope. NIS-Elements D 3.20 image processing software was used for image analysis and data collection to quantify the number of macrophages at the tail injury sites.

### 2.17. Selective Depletion of Macrophages Using Anionic Clodronate Liposomes

For selective and sustained macrophage depletion, anionic clodronate liposomes (Clophosome^®^-A, Formumax, Sunnyvale, CA, USA) were used. Briefly, 150 μL of clodronate liposomes (7 mg/mL) was administered intraperitoneally to 8-week-old male C57BL/6J mice 24 h before palatal wound model establishment. A total of 24 mice were included in the macrophage depletion experiments, comprising two groups (control and 10 μM DHC; *n* = 4 per group per time point). Mice were euthanized on days 0, 3, and 5 after wounding, and palatal wound tissues were collected for subsequent analyses.

### 2.18. Analysis of Blood Routine and Serum Biochemical Parameters

Blood samples from mice were collected into EDTA anticoagulant tubes (for routine blood analysis) or EP tubes (for serum separation). Samples in EP tubes were centrifuged to separate the serum. Blood routine parameters, including white blood cell count (WBC), hemoglobin (HGB), mean corpuscular volume (MCV), mean corpuscular hemoglobin concentration (MCHC), mean platelet volume (MPV), and neutrophil count (NEUT), were detected. Serum biochemical parameters, including alanine aminotransferase (ALT), aspartate aminotransferase (AST), lactate dehydrogenase (LDH), alkaline phosphatase (ALP), and blood urea nitrogen (BUN), were detected using an automatic biochemical analyzer (TOSHIBA TBA-40FR, Tokyo, Japan).

### 2.19. Statistical Analysis

The statistical analysis was carried out using the SPSS 13.0 software package. Prior to analysis, data were evaluated for normal distribution. Student’s *t*-test and one-way ANOVA were utilized to conduct the statistical analysis. The results were presented as the mean ± standard deviation (SD). A significance level of *p* < 0.05 was considered statistically significant. All statistical analyses included a minimum of three biological replicates.

## 3. Results

### 3.1. DHC Inhibits the Chemotactic Function of Macrophages

**Figure 1:**
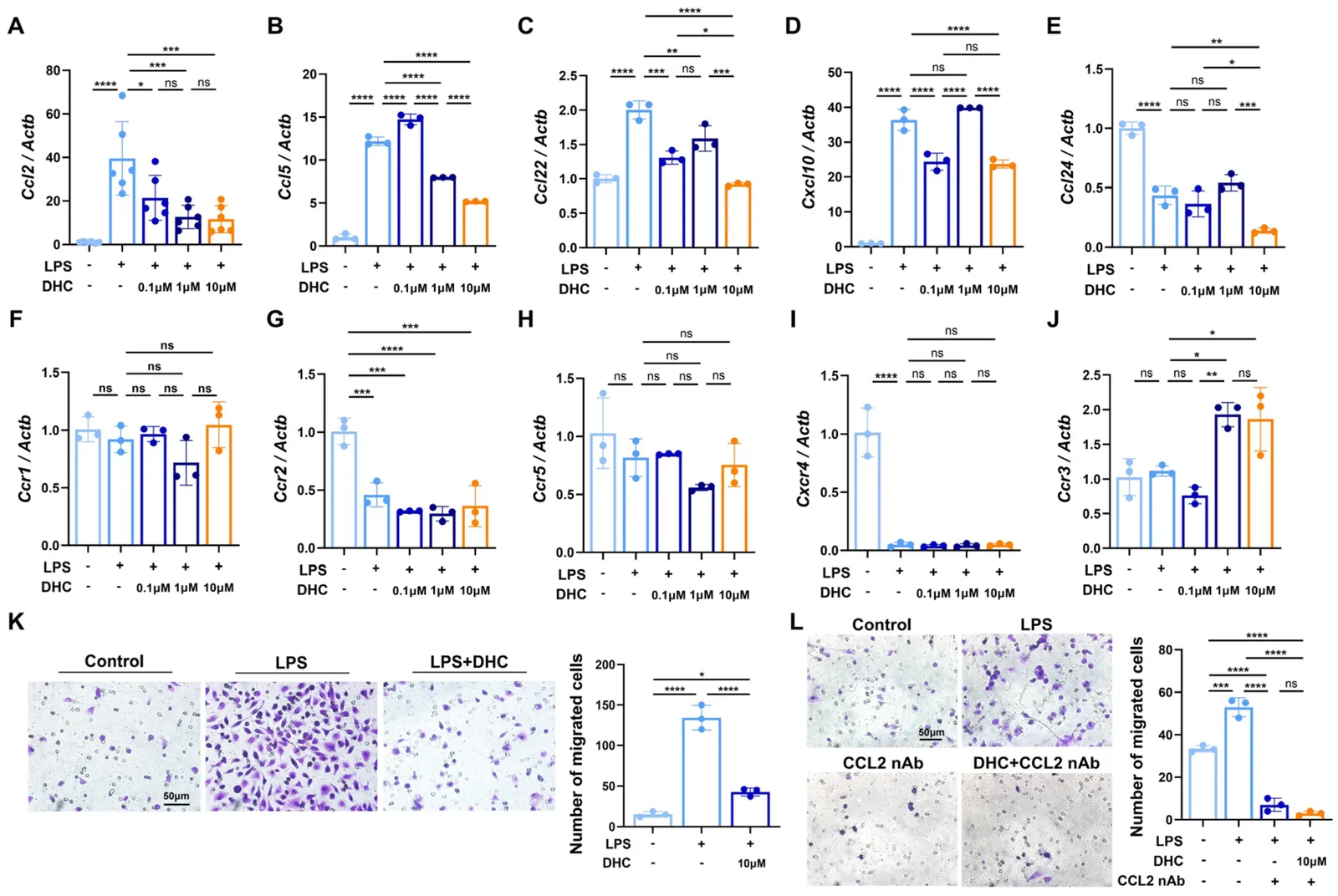
DHC inhibits the chemotactic function of macrophages. (**A**–**J**) Real-time PCR was used to detect the effect of DHC intervention at different concentrations (0.1 μM, 1 μM, and 10 μM) on the expression of chemokines (*Ccl2*, *Ccl5*, *Ccl22*, *Cxcl10*, *Ccl24*) and chemokine receptors (*Ccr1*, *Ccr2*, *Ccr5*, *Cxcr4*, *Ccr3*) in LPS-induced macrophages. (**K**) Representative images from Transwell migration assays (magnification, 100×) showed that treatment with 10 μM DHC for 24 h effectively reversed the LPS-induced increase in the number of migratory primary macrophages. (**L**) Transwell migration assays demonstrated that LPS induction significantly enhanced macrophage migration. However, treatment with CCL2 antibody effectively impeded LPS-induced macrophage migration, while the combined treatment with CCL2 antibody and DHC did not exhibit a further tendency to inhibit macrophage migration. *n* = 6 in (**A**); *n* = 3 in (**B**–**L**). Scale bar: 50 μm in (**K**,**L**). Data in the bar graphs are presented as mean  ±  SD; ns, not significant, *p* > 0.05, * *p* < 0.05, ** *p* < 0.01, *** *p* < 0.001, **** *p* < 0.0001.

DHC treatment (10 μM) broadly reduced chemokine expression in LPS-stimulated macrophages, including *Ccl2* (70.8% reduction), *Ccl5* (57.4% reduction), *Ccl22* (54.1% reduction), *Cxcl10* (34.3% reduction), and *Ccl24* (67.0% reduction), relative to the LPS group (Figure 1A–E). Among these chemokines, *Ccl2* exhibited the most pronounced reduction following DHC treatment and demonstrated a clear dose-dependent response. However, DHC did not significantly alter the expression of chemokine receptors in macrophages (Figure 1F–J). In addition, DHC showed no significant effect on macrophage viability within the tested concentration range (Figure S1A).

This transcriptional suppression was paralleled by functional changes, as DHC reduced LPS-induced macrophage migration by 68.2% relative to the LPS-treated group (Figure 1K). Furthermore, after neutralization of CCL2, DHC failed to produce an additional decrease in macrophage chemotaxis, indicating that the inhibitory effect of DHC on macrophage migration is primarily mediated by CCL2 suppression (Figure 1L).

DHC also attenuated LPS-induced inflammatory activation in macrophages. At the mRNA level, DHC significantly reduced *Il1b* expression (57.3% reduction at 1 μM; 40.9% reduction at 10 μM), *Il6* expression (55.3% reduction at 1 μM), and *Nos2* expression (30.4% reduction at 1 μM; 32.4% reduction at 10 μM), whereas *Tnfa* expression was not significantly affected (Figure S2A–D). In agreement with these transcriptional changes, DHC also significantly reduced the protein abundance of iNOS, IL-1β, and IL-6 (Figure S2E–G). Similar findings were obtained in RAW264.7 cells (Figure S3A–G). Together, these results show that DHC suppresses both the chemotactic behavior and inflammatory activation of M1 macrophages.

### 3.2. DHC Inhibits LPS-Induced Macrophage Chemotaxis by Inhibiting MAPK Signaling

**Figure 2:**
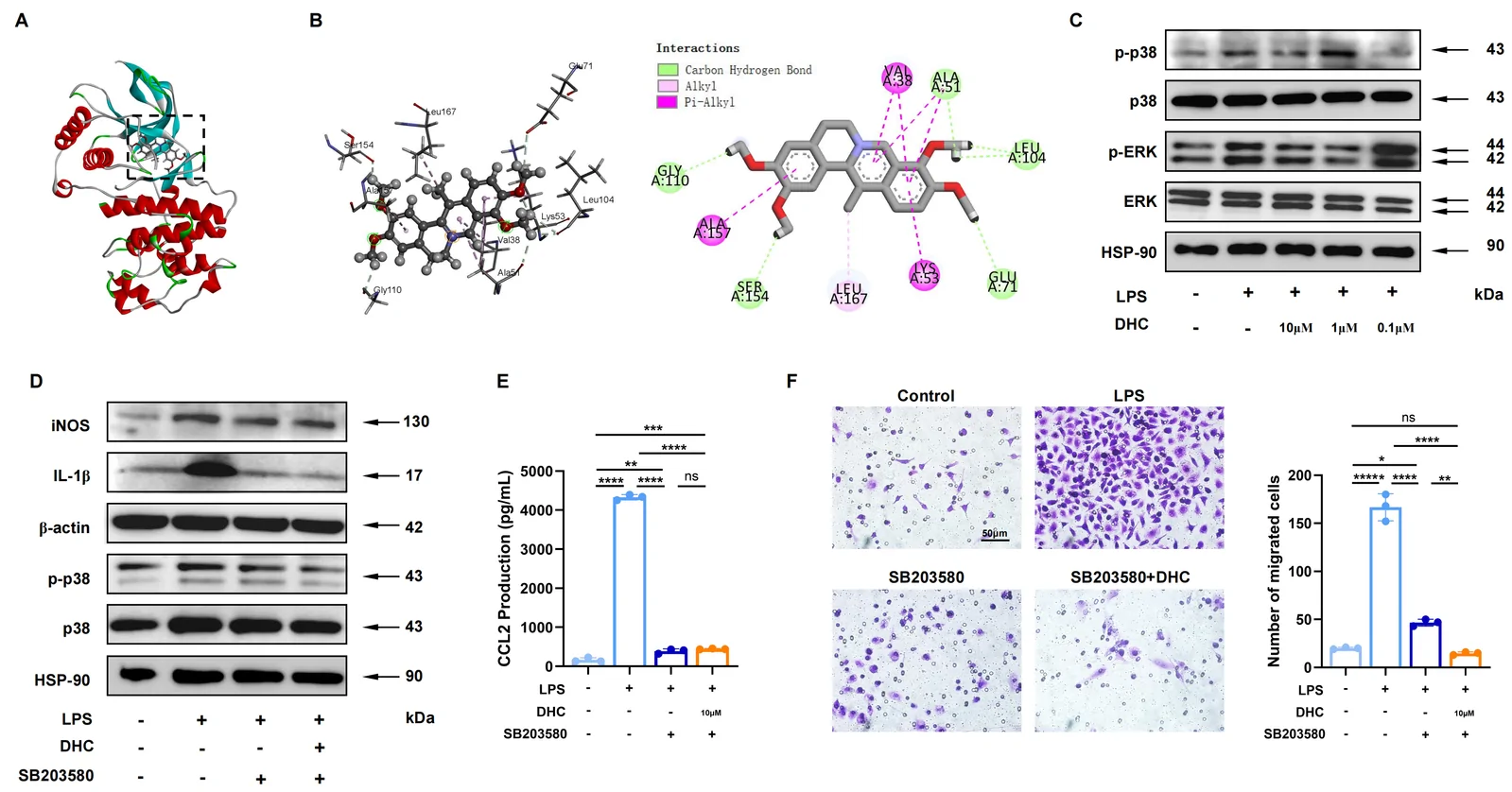
DHC inhibits LPS-induced macrophage chemotaxis by inhibiting MAPK signaling. (**A**,**B**) Molecular docking analysis of the interaction between DHC and MAPK p38 was performed using Discovery Studio 2019 software. (**C**) Western blot analysis revealed a significant decrease in the levels of phosphorylated p38 (p-p38) and phosphorylated ERK1/2 (p-ERK1/2) proteins in macrophages following treatment with 10 μM DHC compared to the control group. (**D**) Western blot was performed to evaluate the effects of SB203580 alone and the combination of SB203580 with DHC on p38 phosphorylation as well as the expression of iNOS and IL-1β. (**E**) ELISA was used to assess the effects of SB203580 alone and the combination of SB203580 with DHC on CCL2 expression. (**F**) The Transwell migration assay was conducted to evaluate the effects of SB203580 alone and the combination of SB203580 with DHC on LPS-induced macrophage chemotaxis. Scale bar: 50 μm in (**F**). Data in the bar graphs are presented as mean  ±  SD; ns, not significant, *p* > 0.05, * *p* < 0.05, ** *p* < 0.01, *** *p* < 0.001, **** *p* < 0.0001.

It has been reported that DHC regulates the inflammatory response of macrophages through the MAPK signaling pathway [17]. To explore whether there is an interaction between DHC and MAPK p38, a key kinase protein in the MAPK pathway, molecular docking simulations of these two molecules were performed in this study. The results showed a Lib Dock score of 100.496, indicating that the key active component (DHC) binds well to the key target (MAPK p38). The root mean square deviation (RMSD) of the molecular docking results was 0.8, suggesting a strong interaction between the small-molecule compound DHC and MAPK p38. These results indicated that DHC may inhibit macrophage chemotaxis by regulating MAPK p38 (Figure 2A,B).

To verify this hypothesis, we performed Western blot analysis to investigate the activation of MAPK signaling following intervention with different concentrations of DHC. The results demonstrated that DHC significantly inhibited the phosphorylation of p38 and ERK1/2 (Figure 2C). Further intervention was performed using SB203580, a selective inhibitor of p38. The Western blot results confirmed that SB203580 significantly inhibited LPS-induced p38 phosphorylation. To further elucidate the molecular mechanism underlying DHC-mediated regulation of macrophage chemotactic function, we used ELISA to assess CCL2 expression in macrophages treated with SB203580. The results revealed a significant downregulation of CCL2 expression upon inhibition of p38 MAPK signaling, and the addition of DHC did not further attenuate CCL2 production (Figure 2E). Similarly, SB203580 alone decreased macrophage migration by 72.0%, whereas the addition of DHC resulted in only a limited further reduction (91.2%) (Figure 2F). These findings suggest that suppression of the p38 MAPK–CCL2 axis accounts for the major inhibitory effect of DHC on macrophage chemotaxis, although the involvement of additional pathways cannot be ruled out.

In addition, we further clarified the impact of DHC-regulated MAPK signaling on the production of inflammatory factors in macrophages. Consistently, inhibition of p38 phosphorylation reversed LPS-induced iNOS and IL-1β expression (Figure 2D). However, co-treatment with SB203580 and DHC failed to further inhibit the expression of iNOS and IL-1β (Figure 2D). Collectively, these results indicate that DHC restrains inflammatory macrophage activation predominantly through inhibition of MAPK signaling.

### 3.3. DHC Significantly Enhances the Healing Process of Palatal Wounds in Mice

**Figure 3:**
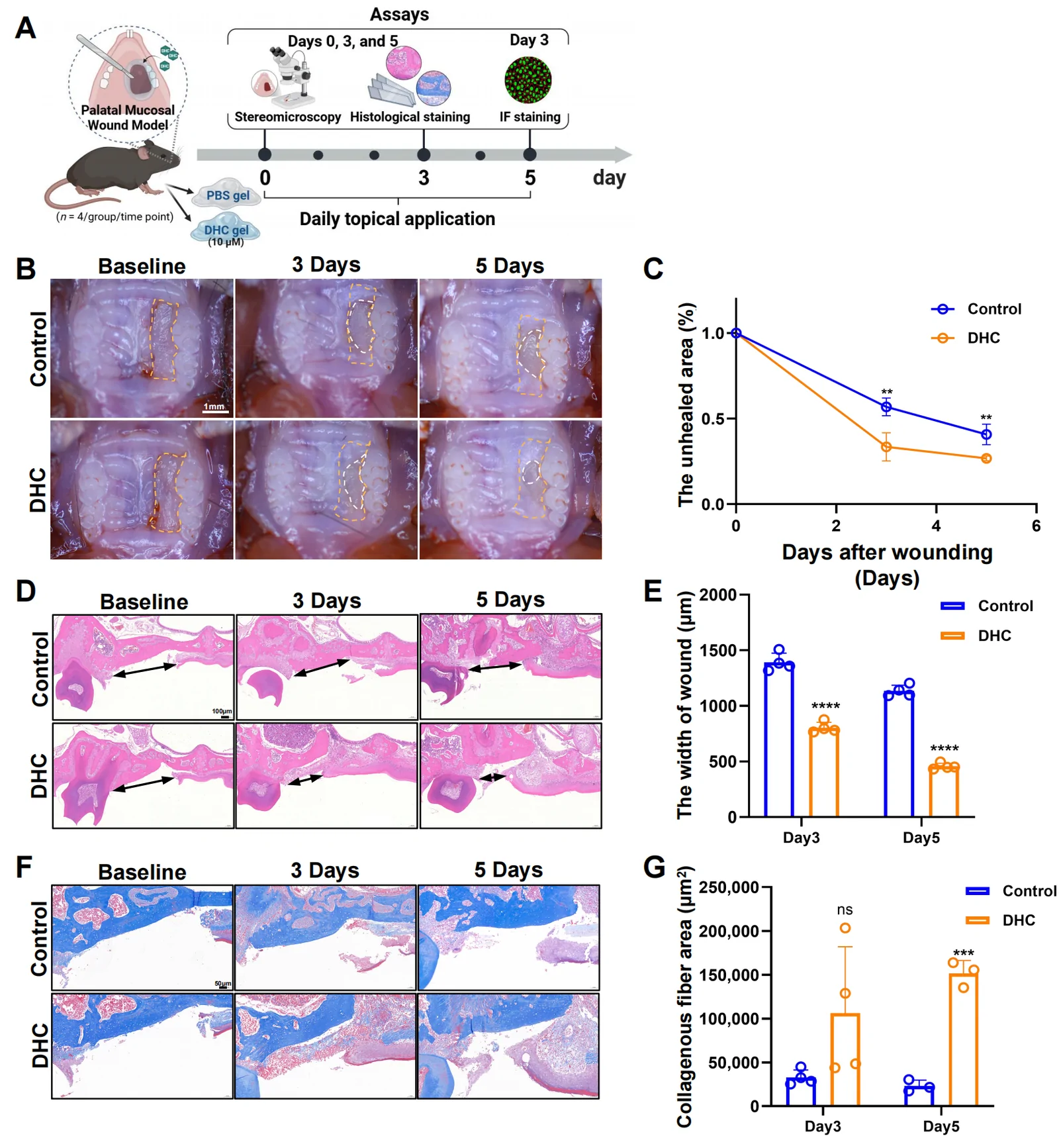
DHC significantly enhances the healing process of palatal wounds in mice. (**A**) Schematic diagram of the mouse palatal mucosal wound model and experimental design. (**B**,**C**) Topical application of DHC significantly improved the palatal wound healing process in mice. The original wound boundary is indicated by yellow dashed lines, and the unhealed wound area is marked by white dashed lines (*n* = 4 per group). (**D**,**E**) H&E staining demonstrated that DHC administration notably accelerated the progression of palatal wound healing in mice on days 3 and 5 after surgery. The width of the unhealed wound is indicated by the black line (*n* = 4 per group). (**F**,**G**) Collagen formation area in palatal wound tissues was detected with Masson’s trichrome staining (day 3: *n* = 4 per group; day 5: *n* = 3 per group). Scale bars: 1 mm in (**B**); 100 μm in (**D**); 50 μm in (**F**). Data in the bar graph are presented as mean  ±  SD; ns, not significant, *p* > 0.05, ** *p* < 0.01, *** *p* < 0.001, **** *p* < 0.0001.

In the mouse palatal wound healing model, topical DHC accelerated palatal mucosal wound closure, with the closure rate reaching 1.6-fold and 1.2-fold of the control on days 3 and 5, respectively (Figure 3A–C). Histopathological analysis further confirmed that DHC treatment markedly promoted epithelial growth and collagen deposition relative to the control group (Figure 3D–G). In addition, topical DHC showed a favorable safety profile, with no obvious abnormalities in routine blood indices, serum biochemical parameters, or histology of major organs after 5 days of treatment (Figure S1B–M). Taken together, these findings indicate that topical DHC safely promotes palatal wound healing and tissue repair.

### 3.4. DHC Influences Palatal Wound Healing by Inhibiting Macrophage Chemotaxis

**Figure 4:**
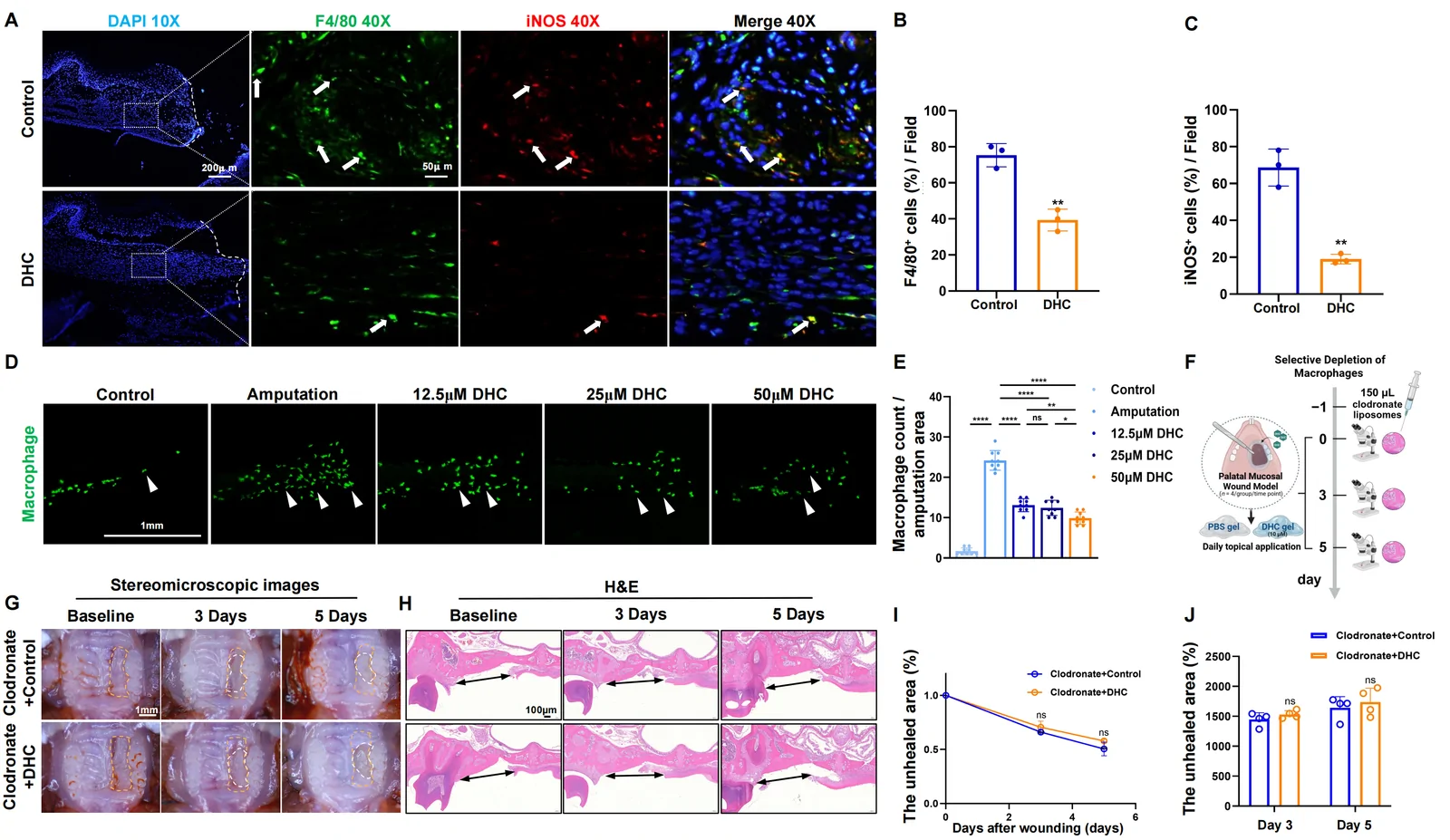
DHC influences palatal wound healing by inhibiting macrophage chemotaxis. (**A**–**C**) Immunofluorescence staining was performed to evaluate the inhibitory effect of DHC on the infiltration of F4/80^+^ and iNOS^+^ macrophages in the regions adjacent to the wound edge. The dashed line denotes the wound edge, and arrows indicate positively stained cells (*n* = 3 per group). (**D**) A zebrafish mechanical injury model was established to examine the effect of DHC at different concentrations (12.5–50 μM) on macrophage chemotaxis during wound healing (*n* = 9 per group). White arrows indicate locally infiltrated macrophages. (**E**) Quantification of macrophage numbers in the tail-amputated region of zebrafish. (**F**) Schematic diagram of the experimental design for selective macrophage depletion in the mouse palatal wound model using anionic clodronate liposomes. (**G**) Representative stereomicroscopic images of palatal wounds in mice at different time points following macrophage depletion with anionic clodronate liposomes (*n* = 4 per group). The yellow dashed line indicates the baseline wound margin, and the white dashed line outlines the unhealed wound area. (**H**) Representative H&E staining of palatal wound tissues (*n* = 4 per group). The black line indicates the width of the unhealed wound. (**I**) Quantification of the unhealed wound area in (**G**) using ImageJ. (**J**) Quantification of wound width in (**H**) measured with ImageJ. Scale bars: 200 μm in (**A**, 10×); 50 μm in (**A**, 40×); 1 mm in (**D**,**G**); 100 μm in (**H**). Data are presented as mean  ±  SD; ns, not significant, *p* > 0.05, * *p* < 0.05, ** *p* < 0.01, **** *p* < 0.0001.

To further explore the impact of DHC on the in vivo inflammatory response in wounds, immunofluorescence staining was performed on palatal wound tissues at 3 days post-modeling. The results demonstrated that DHC administration effectively inhibited the infiltration of F4/80^+^ macrophages (47.4% reduction) and reduced the number of iNOS^+^ M1 macrophages (72.3% reduction) in the wound area (Figure 4A–C). Consistent results were obtained in the zebrafish mechanical injury model, where DHC attenuated macrophage recruitment to the injured area in a concentration-dependent manner (12.5 μM, 45.2% reduction; 25 μM, 48.3% reduction; 50 μM, 58.8% reduction) relative to the injured control (Figure 4D,E).

Importantly, following selective macrophage depletion, DHC no longer enhanced wound closure, and the healing rates remained comparable between the DHC-treated and control groups (Figure 4F–J). These results support the interpretation that the wound-healing effect of DHC is largely dependent on its ability to limit macrophage chemotaxis.

### 3.5. DHC Suppresses the Inflammatory Response Within the Wound

**Figure 5:**
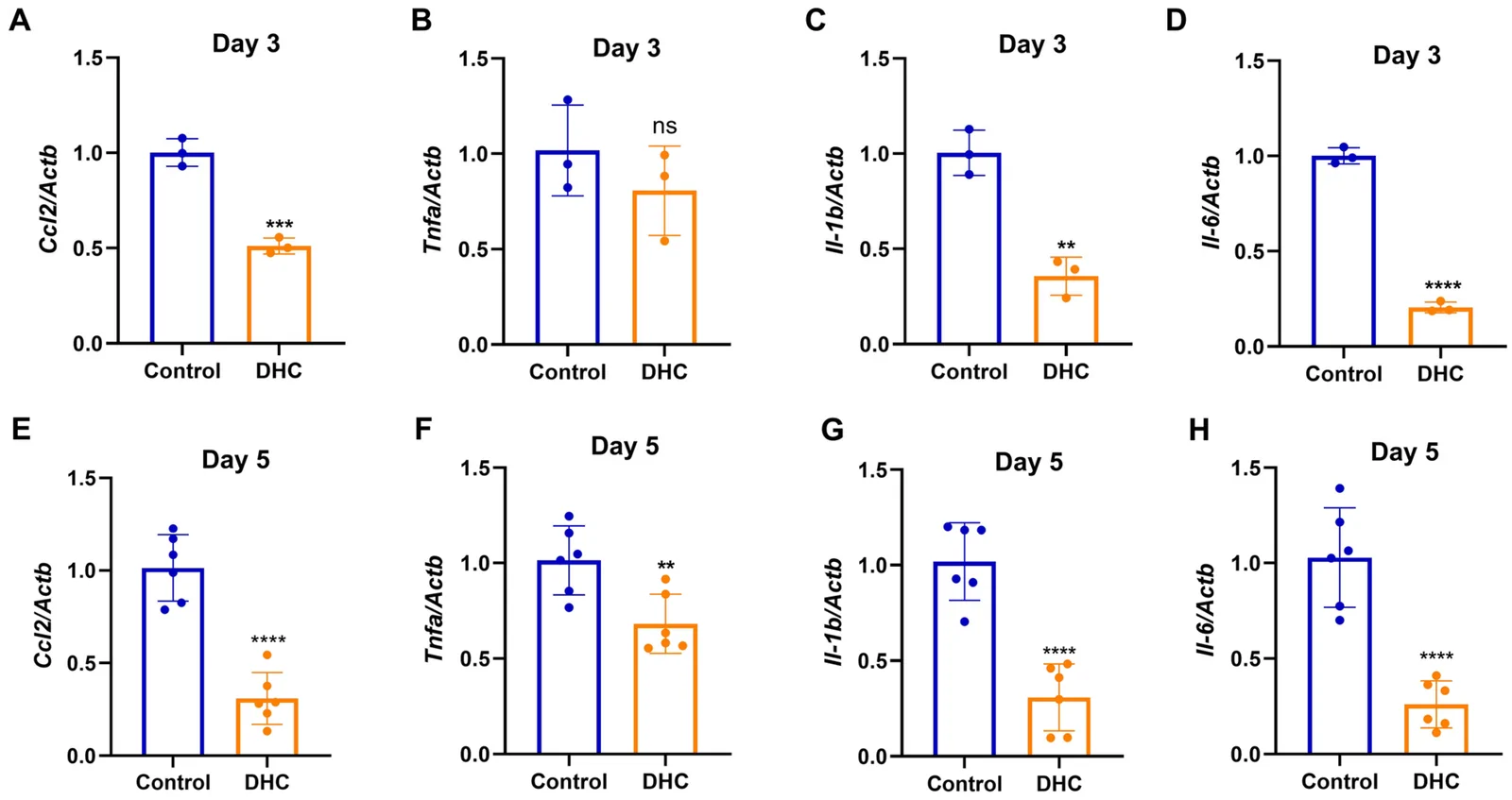
DHC suppresses the inflammatory response in the wound. (**A**–**D**) Real-time PCR was performed to detect the effect of 10 μM DHC gel on the expression of *Ccl2, Il6*, *Il1b*, and *Tnfa* on day 3 of wound healing (*n* = 3 per group). (**E**–**H**) Real-time PCR analysis revealed that topical application of 10 μM DHC gel effectively suppressed the expression of *Ccl2, Il6*, *Tnfa*, and *Il1b* on day 5 of wound healing (*n* = 6 per group). Data in the bar graphs are presented as mean  ±  SD; ns, not significant, *p* > 0.05, ** *p* < 0.01, *** *p* < 0.001, **** *p* < 0.0001.

To further evaluate the effect of DHC on local wound inflammation, wound tissues were collected on days 3 and 5 for real-time PCR analysis. The results revealed that DHC reduced *Ccl2* mRNA expression in wound tissues at both time points examined, with reductions of 48.9% on day 3 and 67.8% on day 5 relative to the control group (Figure 5A,E). Consistently, the expression of pro-inflammatory cytokines *Il6*, *Il1b*, and *Tnfa* in wound tissues was also significantly decreased (Figure 5B–D,F–H). Overall, these results suggest that DHC suppresses *Ccl2* expression within the wound microenvironment, thereby limiting macrophage recruitment and preventing the amplification of the inflammatory response during healing.

## 4. Discussion

Inflammation establishes a microenvironment conducive to microbial dysbiosis, forming a self-perpetuating cycle that ultimately impairs wound resolution. Throughout the wound-healing process, macrophages exhibit pronounced phenotypic plasticity and perform context-dependent functions in the regulation of inflammation and tissue repair [19,20]. Therefore, pharmacological modulation of this inflammatory phase has emerged as a promising strategy to improve healing outcomes. Li et al. demonstrated that SZC-6, a coumarin derivative, accelerated diabetic wound closure by suppressing NF-κB-mediated macrophage inflammation [21]. Similarly, topical application of *Resina Draconis* has been shown to promote wound healing through attenuation of oxidative stress and regulation of macrophage polarization [22]. Although previous studies have reported the anti-inflammatory effects of DHC in atherosclerosis [17], its role in oral mucosal wound healing has not been investigated. In the present study, we found that DHC accelerated palatal wound healing and reduced macrophage infiltration in wound tissues. Mechanistically, these effects may be associated with impaired macrophage chemotaxis through modulation of the p38 MAPK/CCL2 axis. These findings expand the known pharmacological profile of DHC and suggest that CCL2 may be a potential downstream mediator of its pro-healing effects during palatal wound healing.

Chemokines, a family of chemotactic cytokines, are essential mediators of wound healing that regulate the timely recruitment of immune cell subsets to sites of tissue injury [23]. Chemokine production is induced in both innate and adaptive immune cells in response to tissue damage [24,25]. Dysregulated chemokine expression may contribute to prolonged inflammation in chronic wounds and other pathological conditions. Pang et al. demonstrated that sustained CCL2/CCR2 signaling in diabetic wounds maintained a pro-inflammatory macrophage phenotype and delayed wound healing [26]. In addition, Park et al. reported that the therapeutic peptide AESIS-1 accelerated wound healing by promoting fibroblast migration in a CXCR2-dependent manner [27]. Collectively, these studies underscore the importance of chemokine signaling in orchestrating wound repair. Consistent with these observations, our data suggest that macrophages contribute to the pro-healing effects of DHC. In vitro, DHC inhibited CCL2-dependent macrophage chemotaxis, and in vivo, DHC reduced macrophage infiltration in the wound area and accelerated palatal wound healing. Notably, the beneficial effect of DHC on wound healing was abolished following macrophage depletion, suggesting that macrophages are involved in this process, at least in part. A potential mechanism is that DHC suppresses *Ccl2* expression within the wound microenvironment, thereby limiting macrophage infiltration and attenuating local inflammation. Such changes may help establish a microenvironment that favors the transition from the inflammatory phase to the proliferative phase of healing, thereby facilitating tissue repair. However, the molecular basis underlying this effect remains to be further elucidated.

The MAPK family, which includes JNK, ERK, p38, and other kinases, serves as a central hub for transducing extracellular stimuli into intracellular inflammatory and migratory responses [28]. It is well established that LPS activates MAPK signaling via TLR4, thereby upregulating pro-inflammatory mediators such as iNOS, IL-1β, and IL-6 and amplifying macrophage inflammatory responses [29]. Our data indicate that DHC inhibits macrophage chemotaxis in vitro primarily through the p38 MAPK/CCL2 axis. This mechanism may provide a molecular basis for our in vivo observations that DHC suppressed macrophage infiltration in wound tissues and accelerated palatal wound healing. This observation is consistent with the findings of Hu et al., who reported that DHC inhibited the MEK1/2-ERK1/2 cascade in melanoma cells [30]. Recent studies have further underscored the therapeutic relevance of targeting the MAPK pathway in inflammatory conditions. For instance, Hu et al. demonstrated that the Cimicifugae Rhizoma/*Smilax glabra* Roxb. herb pair could attenuate chronic inflammatory skin diseases by downregulating MAPK pathway-mediated expression of cytokines and chemokines, while simultaneously improving amino acid and carnitine metabolism in vivo [31]. Similarly, Xia et al. reported that Corydalis-derived protoberberines exhibited anti-inflammatory activity through modulation of the MAPK and NF-κB signaling pathways [18]. Collectively, these findings support the view that the p38 MAPK/CCL2 axis is a key mechanistic pathway mediating the anti-inflammatory and anti-chemotactic effects of DHC in macrophages. Nevertheless, DHC may also influence other inflammatory pathways, including NF-κB signaling, as reported by Wen et al. [17]. The relative contribution of these pathways to the overall pro-healing effects of DHC remains to be elucidated.

## 5. Conclusions

In conclusion, DHC reduced *Ccl2* expression by 70.8%, primarily via the MAPK pathway, thereby inhibiting macrophage chemotaxis. Consistent with these effects, DHC suppressed macrophage infiltration and attenuated inflammatory responses during the early stage of wound healing. Moreover, DHC accelerated wound healing by 1.6-fold. These findings support DHC as a promising anti-inflammatory small molecule for mucosal repair. Nevertheless, further studies incorporating pathway rescue and genetic validation approaches would help to more firmly define the mechanistic links among DHC treatment, p38 MAPK inhibition, and reduced CCL2 expression. In addition, validation in human cells, together with studies in additional experimental models and longer-term assessments of wound remodeling and scar formation, would further strengthen these findings. Overall, DHC may provide an effective therapeutic strategy for inflammation-associated oral mucosal wounds.

## Data Availability

The datasets used and/or analyzed during the current study are available from the corresponding author on reasonable request. The data are not publicly available due to [laboratory data management policies].

## Supplementary Materials

The following supporting information can be downloaded at: https://www.mdpi.com/article/10.3390/biomedicines14091918/s1, Figure S1: *In vitro* and *in vivo* biosafety assessment of DHC; Figure S2: DHC inhibits the expression of inflammatory genes in macrophages; Figure S3: DHC inhibits inflammatory function in RAW264.7 macrophages; Table S1: The antibodies and reagents used in this experiment; Table S2: Primers sequences used in the real-time PCR.

## Abbreviations

The following abbreviations are used in this manuscript:

ALP	alkaline phosphatase
ALT	alanine aminotransferase
ARRIVE	Animal Research: Reporting of In Vivo Experiments
AST	aspartate aminotransferase
BUN	blood urea nitrogen
CCL	C-C motif chemokine ligand
CXCL	C-X-C motif chemokine ligand
DHC	dehydrocorydaline
dpf	days post-fertilization
ELISA	enzyme-linked immunosorbent assay
FBS	fetal bovine serum
H&E	hematoxylin and eosin
HGB	hemoglobin
LDH	lactate dehydrogenase
LPS	lipopolysaccharide
MAPK	mitogen-activated protein kinase
MCHC	mean corpuscular hemoglobin concentration
MCV	mean corpuscular volume
MPV	mean platelet volume
MTC	maximum tolerated concentration
NEUT	neutrophil count
SD	standard deviation
WBC	white blood cell count
