# Supplementary material for: Dehydrocorydaline Accelerates Palatal Wound Healing in Mice Through Suppression of the p38 MAPK/CCL2 Axis and Macrophage Chemotaxis: A Preliminary Study
Source: Biomedicines. 2026 Aug 27;14(9):1918. doi: 10.3390/biomedicines14091918 (PMC13604338; doi:10.3390/biomedicines14091918)
Supplement: Supplementary file 1 [file biomedicines-14-01918-s001.zip › Supplementary figure.pdf]

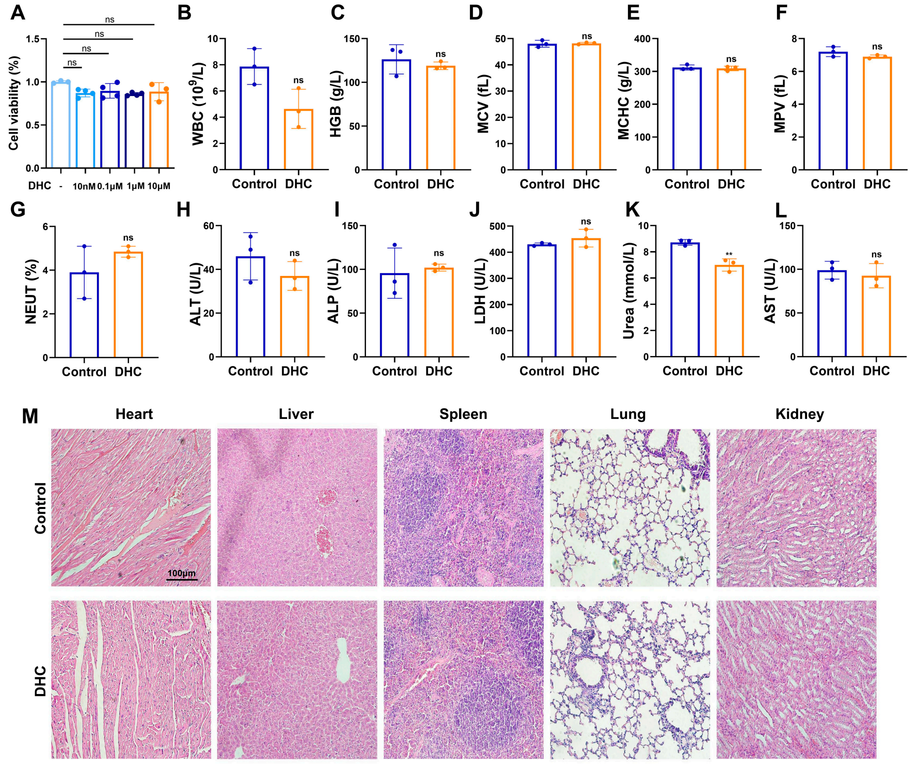

Supporting Figure S1 *In vitro* and *in vivo* biosafety assessment of DHC. (A) Viability of RAW264.7 cells after treatment with DHC at the indicated concentrations (10 nM, 0.1 μM, 1 μM, and 10 μM) for 24 h, as determined by the CCK-8 assay. (B-L) Blood routine and serum biochemical analyses in mice treated with DHC or vehicle control, including WBC (B), HGB (C), MCV (D), MCHC (E), MPV (F), NEUT (G), ALT (H), ALP (I), LDH (J), Urea (K), and AST (L). (M) Representative H&E-stained sections of major organs, including heart, liver, spleen, lung, and kidney, collected from control and DHC-treated mice. Scale bar = 100 μm. Data are presented as means ± SD; ns, not significant, $P > 0.05$, $^{**}P<0.01$.

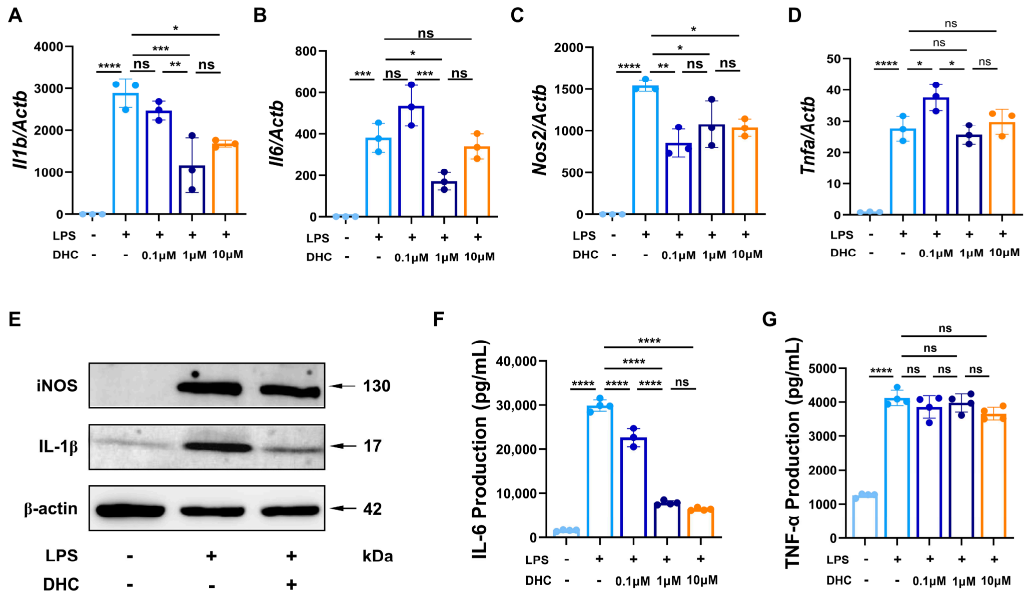

Supporting Figure S2 DHC inhibits the expression of inflammatory genes in macrophages. (A-D, F, G) Mouse peritoneal macrophages were treated with LPS and the indicated concentrations of DHC (0.1, 1, and 10 μM) for 24 h. (A-D) Real-time PCR analysis was performed to evaluate the expression levels of inflammatory genes (*Il1b*, *Il6*, *Nos2*, and *Tnfa*). (E) Western blot analysis of iNOS and IL-1β protein expression, with β-actin as a loading control. (F, G) ELISA results demonstrated the protein expression levels of inflammatory factors IL-6 and TNF-α. All experiments were independently replicated three times. Data in the bar graphs are presented as means ± SD; ns, not significant, P>0.05, *P<0.05, **P<0.01, ***P<0.001, ****P<0.0001.

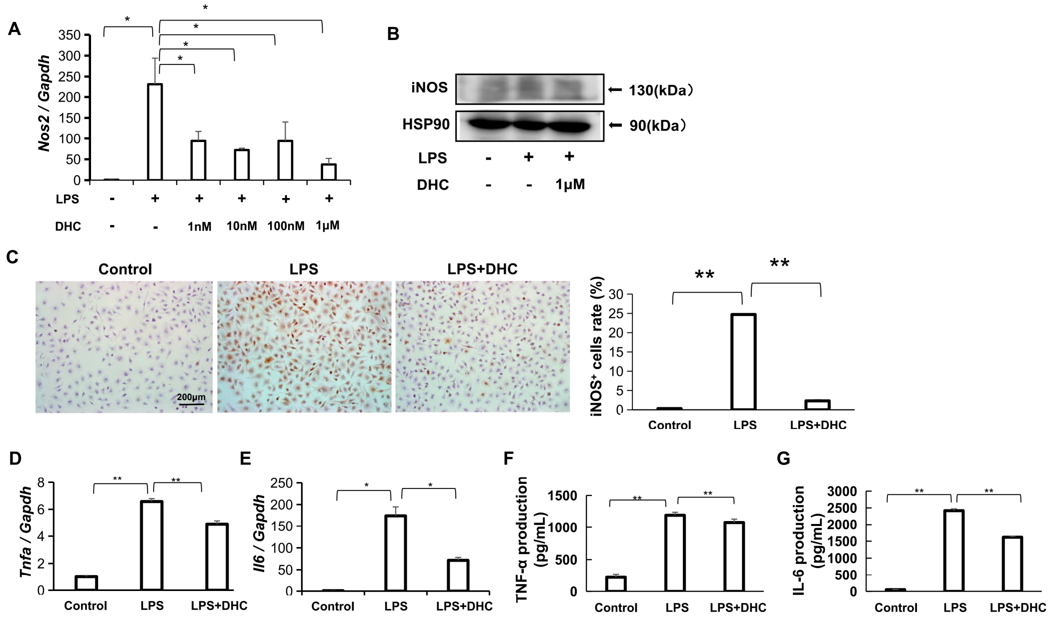

Supporting Figure S3 DHC inhibits inflammatory function in RAW264.7 macrophages. (A)

Real-time PCR analysis of *Nos2* mRNA expression in RAW264.7 macrophages after LPS stimulation and DHC treatment. (B) Western blot analysis of iNOS protein expression in RAW264.7 macrophages after LPS stimulation and DHC treatment. HSP90 was used as a loading control. (C) Representative immunostaining images and quantification of iNOS-positive RAW264.7 macrophages in the control, LPS, and LPS + DHC groups. (D, E) Real-time PCR analysis of *Tnfa* and *Il6* mRNA expression in RAW264.7 macrophages after LPS stimulation and DHC treatment. (F, G) ELISA analysis of TNF-α and IL-6 production in RAW264.7 macrophages after LPS stimulation and DHC treatment. All the above results were independently replicated three times. All experiments were independently repeated three times. Scale bar: 200 μm in (C). Data in the bar graphs are presented as means ± SD; $^*P<0.05$, $^{**}P<0.01$.
